# Toxicological evaluation of a pumpkin-derived pectin preparation: in vitro genotoxicity studies and a 13-week oral toxicity study in Sprague-Dawley rats

**DOI:** 10.1093/toxres/tfae004

**Published:** 2024-01-23

**Authors:** Anne F Kleijn, Margien Mutter, James A Akingbasote, Jwar Meetro, Ryan R Simon, Pieter Muntendam, Matthias Frommhagen, Henk A Schols

**Affiliations:** Laboratory of Food Chemistry, Wageningen University and Research, Bornse Weilanden 9, Wageningen, WG 6708, The Netherlands; G3P Inc., 20 Mall Road Suite 220, Burlington, MA 01803, United States; Intertek Health Sciences Inc., Food and Nutrition Group, 2233 Argentia Road, Suite 201, Mississauga, ON L5N 2X7, Canada; Intertek Health Sciences Inc., Food and Nutrition Group, 2233 Argentia Road, Suite 201, Mississauga, ON L5N 2X7, Canada; Intertek Health Sciences Inc., Food and Nutrition Group, 2233 Argentia Road, Suite 201, Mississauga, ON L5N 2X7, Canada; G3P Inc., 20 Mall Road Suite 220, Burlington, MA 01803, United States; Société des Produits Nestlé SA, Nestlé Research, Route du Jorat 57, CH-1000, Lausanne 26, Switzerland; Laboratory of Food Chemistry, Wageningen University and Research, Bornse Weilanden 9, Wageningen, WG 6708, The Netherlands

**Keywords:** pectin, pumpkin, genotoxicity, subchronic toxicity, safety, Galectin-3, RG-I

## Abstract

The safety of a rhamnogalacturonan-I-enriched pectin extract (G3P-01) from pumpkin (*Cucurbita moschata* var. Dickinson) was evaluated for use as an ingredient in food and dietary supplements. G3P-01 was tested in a battery of genetic toxicity studies including reverse mutagenicity and *in vitro* micronucleus assay. In addition, Sprague-Dawley rats were randomized and orally dosed with G3P-01 incorporated in animal diet at concentrations of 0, 9000, 18,000, and 36,000 ppm daily for 13-weeks (n=10/sex/group) in line with OECD guidelines (TG 408). The results of the *in vitro* bacterial reverse mutation assay and micronucleus assay in TK6 cells demonstrated a lack of genotoxicity. The 13-week oral toxicity study in Sprague-Dawley rats demonstrated that the test article, G3P-01 was well tolerated; there were no mortalities and no adverse effects on clinical, gross pathology, hematology, blood chemistry, and histological evaluation of the essential organs of the animals. The present study demonstrates that G3P-01 is non-genotoxic and is safe when ingested in diet at concentrations up to 36, 000 ppm. The subchronic no-observed-adverse-effect level (NOAEL) for G3P-01 was concluded to be 36,000 ppm, equivalent to 1,899 and 2,361 mg/kg/day for male and female rats respectively.

## Introduction

**Fig. 1:**
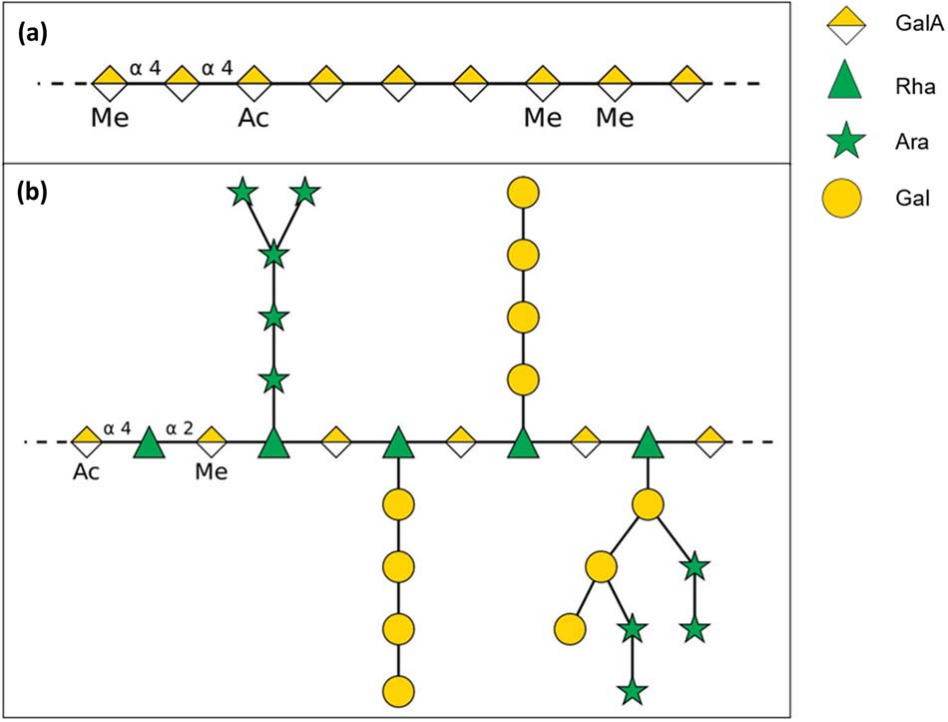
Schematic overview of the structure of (a) pectic homogalacturonan and (b) rhamnogalacturonan-I, which are the main structural elements of native pectin as present in fruit and vegetables. Ac = acetyl, Me = methyl structure based on Waldron et al.^71^, created with DrawGlycan (Cheng et al.^72^).

Galectin-3 is considered an important mediator of cardiac, renal and neurodegenerative disorders and certain fibrotic diseases affecting lung and liver.^1–3^ Consequently Galectin-3 inhibition is widely viewed as offering a potential path to prevention or treatment of galectin-3 mediated conditions^1–5^ Pectins have been shown to bind to the carbohydrate recognition domain (CRD) of galectin-3^6^ and pectin binding to the CRD is associated with disease modifying effects across species and animal models.^3^^,^^7–11^ Pectin is a structurally and functionally complex polysaccharide in plants and is associated with cell wall components including (hemi)cellulose and lignin.^12^ Native pectin is composed of structural elements with varying monosaccharide composition, branching, and esterification. The most abundant component of pectin is homogalacturonan (HG), which is composed of galacturonic acid (GalA) residues; GalA residues exist in methyl esterified and/or acetylated states.^13^ Another component of pectin, rhamnogalacturonan-I (RG-I), is a heteropolymer consisting of a backbone of alternating rhamnose and GalA residues with arabinose- and/or galactose-containing sidechains, and possible *O*-methyl- and acetyl esterified GalA residues^14^^,^^15^ (Fig. 1).

RG-I can be released from pectin via enzymatic hydrolysis or chemical degradation of non–RG-I pectin components such as HG.^16–18^ The structure of pectin isolated from plants may differ depending on plant species, developmental stage, and method of extraction. These factors can affect the monosaccharide composition, size distribution, as well as degree of acetyl and methyl esterification of pectin.^19–21^

Commercial pectin preparations are rich in HG and are primarily derived from citrus fruit peels and apple pomace, as well as potatoes, carrots, and sugar beets.^22–24^ Pectin ingredients are hydrocolloids and typically used in food for their gelling and thickening properties.^25^ Pectin is consumed in the diet as a constituent of fruits and vegetables, as well as through processed foods that contain added pectin ingredients. The pectin content in fruits and vegetables is estimated to be 0.59% and 0.66% fresh weight, respectively.^26^ Pectins are a class of dietary fiber and have been reported to have beneficial effects on blood lipid control,^27^ immunomodulation, and cardiac fibrosis.^3^^,^^28^ As prebiotics, pectins are indigestible by human digestive enzymes but are fermented by beneficial microbiota in the large intestine to produce short-chain fatty acids (SCFAs).^29^ SCFAs have been suggested to play a role in the reduction of obesity,^30^ diabetes, and mucosal inflammation,^31^ among others.

G3P-01 is an RG-I-enriched pectin extract obtained from pumpkin (*Cucurbita moschata* var. Dickinson) specifically developed for its galectin-3 binding activity. It has been developed for use as an ingredient in food and dietary supplements to supplement dietary pectin intake from fruits and vegetables in the diet. G3P-01 is extracted from pumpkin using food-grade commercial enzyme preparations for the degradation of especially starch and pectic homogalacturonan.

The current study was designed to evaluate the safety of G3P-01 using genotoxicity and oral toxicity models in order to meet the global regulatory standards established for the safety of ingredients used in dietary supplements and food. To determine the genotoxicity potential of G3P-01, a bacterial reverse mutation (Ames) assay and an in vitro micronucleus assay in TK6 cells were conducted. Further, systemic toxicity of the extract was evaluated using animal studies by means of a 14-day dose range–finding study and a 13-week repeat-dose subchronic toxicity study with a 28-day recovery.

## Materials and methods

Studies were conducted at Charles River Laboratories (Ashland, USA) in compliance with Good Laboratory Practice (GLP).^32^ The bacterial reverse mutation (Ames) assay was conducted according to Organisation for Economic Co-operation and Development (OECD) Test Guideline 471 (*Bacterial Reverse Mutation Test*^33^), while the micronucleus assay in TK6 cells was conducted according to OECD Test Guideline 487 (In Vitro *Mammalian Cell Micronucleus Test*^34^). The 13-week repeat-dose subchronic toxicity study was conducted according to OECD Test Guideline 408 (*Repeated Dose 90-Day Oral Toxicity Study in Rodents*^35^). All animal studies were conducted according to the most recent *Guide for the Care and Use of Laboratory Animals* published by the National Research Council.^36^

### Materials

G3P-01 is produced from pumpkin (*C. moschata*) using food-grade commercial enzyme mix containing α-amylase and amyloglucosidase, cellulases and pectinases); this is followed by subsequent filtration steps. The final product is freeze-dried to a powder containing pectins with a molecular weight distribution of 30-70 KDa. Batches of G3P-01 (Batch Nos. 200268 and 200419) were provided by G3P Inc. (Burlington, USA) and were used for the 13-week subchronic oral toxicity study; only Batch No. 200268 was used for the bacterial reverse mutation test and in vitro micronucleus assay. Analytical data of the composition and heavy metal and microbiological contaminants are presented in Table 1. G3P-01 is largely composed of carbohydrates (56–57 (%w/w)), fat (18–21 (%w/w)) protein (8-10 (%w/w)) and does not contain heavy metals or microbiological contaminants at levels that would pose safety concerns.

**Table 1:** Composition of rhamnogalacturonan-I-enriched pectin extract. (G3P-01) for Batch Nos. 200268 and 200418 obtained from our institution used in the current study.

Parameter	G3P-01 Batch No. 200268	G3P-01 Batch No. 200419
Proximate parameters (%w/w)		
Total carbohydrates (%w/w)	56.0	57.4
Total fatty acids	17.5	14.75
Protein (%w/w)	7.8	9.6
Ash (% w/w)	3.86	3.92
Moisture (% w/w)	1.81	2.44
Amino acids (%w/w)		
Serine	0.536	0.653
Glutamic acid	1.3	1.41
Proline	0.394	0.425
Glycine	0.379	0.389
Alanine	0.651	0.739
Valine	0.493	0.589
Isoleucine	0.253	0.309
Leucine	0.471	0.568
Tyrosine	0.204	0.267
Phenylalanine	0.243	0.319
Lysine	0.507	0.571
Histidine	0.131	0.165
Arginine	0.336	0.33
Hydroxyproline	0.357	0.35
Threonine	0.425	0.551
Aspartic acid	0.611	0.733
Ornithine	<0.05	<0.05
Tryptophan (total)	0.117	0.167
Methionine	0.087	0.094
Cysteine + cystine	0.076	0.095
Fatty Acids (%w/w G3P-01)		
Lauric acid	0.03	0.03
Myristic acid	0.04	0.04
Palmitic acid	3.96	3.43
Hexadecenoic Acid	0.02	0.02
Margaric acid	0.02	0.02
Stearic acid	0.95	0.70
Vaccenic Acid	0.03	-
Octadecenoic Acid	0.13	0.12
Oleic Acid	2.42	1.70
Linoleic acid	7.34	5.93
Conjugated Linoleic Acid	0.07	0.07
alpha-Linolenic acid	2.25	2.48
Arachidic acid	0.06	0.05
Gondoic acid [C20:1n-9t]	0.01	0.01
Gondoic acid [C20:1 (n-9c)]	0.01	-
Eicosatetraenoic Omega 6	0.02	0.02
Eicosapentaenoic (EPA)	-	0.01
Behenic acid	0.04	0.04
Tricosanoic acid	0.04	0.03
Lignoceric acid	0.06	0.05
Total	17.5	14.75
Monosaccharides (anhydrous weight) (% w/w G3P-01)		
Fucose	0.2	0.2
Arabinose	5.8	5.7
Rhamnose	6.0	5.8
Galactose	23.9	23.8
Glucose	5.6	7.0
Xylose	0.3	0.3
Mannose	0.6	0.7
Uronic acids	13.6	14.0
acetyl-groups	1.3	1.5
methyl-esters	0.1	0.12
Minerals		
Calcium	8,900	6,400
Iron	85	100
Magnesium	2,400	2,300
Phosphorus	3,200	3,300
Phosphorus pentoxide	7,300	7,600
Phosphate	9,800	10,000
Potassium	5,200	8,300
Sodium	670	710
Heavy metals (mg/kg)		
Aluminum	2.5	9.1
Arsenic	<0.040	<0.040
Cadmium	<0.020	<0.020
Copper	2.1	2.5
Lead	0.055	0.047
Manganese	20	18
Mercury (μg/kg)	<2.0	<2.0
Nickel	0.11	<0.080
Tin	<0.040	<0.040
Zinc	15	12
Microbiological parameters (CFU/g)		
Aerobic mesophilic count	<10	<10
Yeasts	<10	<10
Molds	<10	<10
*Bacillus cereus*	<100	<100
Enterobacteriaceae	<10	<10
*Escherichia coli*	<10	<10
*Salmonella* (in 25 g)	Absent	Absent
Coagulase positive *Staphylococcus*	<100	<100
Heat-resistant mesophilic spore count	<10	<10
Heat-resistant thermophilic spore count	<10	<10
Spore-forming anaerobe mesophilic count	<10	<10
Spore-forming anaerobe thermophilic count	<10	<10

CFU = colony-forming units.

### Methods

#### Bacterial reverse mutation test

Solutions of G3P-01 from Batch No. 200268 were prepared in sterile water (Corning; Manassas, USA). Four strains of *Salmonella typhimurium* (TA98, TA100, TA1535, and TA1537) and 1 *Escherichia coli* strain (WP2 *uvrA*), (Molecular Toxicology Inc., Boone, USA), were used for mutagenicity testing. Prior to each test day, an inoculum from a frozen clone of each strain was incubated overnight for approximately 8.25 to 9 h in Oxoid nutrient broth at 36 to 38 °C with shaking. Optical density readings were performed spectrophotometrically at 650 nm on all strains to check the level of growth. Viable cell titers were also performed in the mutagenicity assay, and 10^8^ to 10^9^ cells per mL of culture were used for all strains.

In the concentration range-finding assay, G3P-01 was tested in strains TA100 and WP2 *uvrA* at 0, 1, 5, 10, 50, 100, 500, 1,000, and 5,000 μg/plate with and without metabolic activation using the plate incorporation method. In the main mutagenicity assay, G3P-01 was tested at 0, 25, 50, 100, 250, 500, 1,000, and 2,500 μg/plate with and without metabolic activation using the plate incorporation method. A top concentration 2,500 μg/plate was chosen as there were precipitates formed which would interfere with interpretation of the result at 5,000 μg/plate. Sterile water was used as a control for all assays. Testing was performed in triplicate for each G3P-01 concentration with and without metabolic activation.

For tests without metabolic activation, 2.5 μg/plate 2-nitrofluorene (Sigma-Aldrich Inc.; Saint Louis, USA) was used for strain TA98; 1.0 μg/plate sodium azide (Sigma-Aldrich Inc.; Saint Louis, USA) was used for strains TA100 and TA1535; 0.5 μg/plate ICR-191 acridine (Sigma-Aldrich Inc.; Saint Louis, USA) was used for strain TA1537; and 2.0 μg/plate 4-nitroquinoline-N-oxide (Acros Organics; Geel, Belgium) was used for strain WP2 *uvrA*. For tests with metabolic activation, 2-aminoanthracene (Sigma-Aldrich Inc.; Saint Louis, USA) was used as the positive control for all strains (2.5 μg/plate for TA98, TA100, TA1535, and TA1537; 10 μg/plate for WP2 *uvrA*)*.*

For metabolic activation, microsomal fractions (S9) from the livers of male rats induced with phenobarbital and 5,6-benzoflavone were prepared and used as described in Ames et al.^37^ and Maron and Ames.^38^ The 9,000 × g liver S9, glucose-6-phosphate, and MgCl_2_-KCl in 0.1 M phosphate buffer (“Regensys A”) and the lyophilized nicotinamide adenine dinucleotide phosphate (“Regensys B”) (all from Molecular Toxicology Inc.; Boone, USA) were combined to create the metabolic activation mixture (S9). The mixture was maintained at 2 to 8 °C or on ice prior to use, on the day of testing. Regensys A and Regensys B were removed from storage and maintained at ambient temperature. Regensys A was maintained at 2 to 8 °C or on ice. Regensys A was used to reconstitute an appropriate aliquot of Regensys B. The Regensys A and B solutions were mixed prior to formulating the S9 mixture. The S9 mixture contained 7.5% (v/v) S9.

For plate incorporation, sterile 12 × 75 mm test tubes were placed in heating blocks at approximately 46 °C and the following items were added in a stepwise manner for each concentration of test or control article: 2.00 mL of top agar, supplemented with 10% of a 0.5-mM histidine/biotin/tryptophan solution; 0.10 mL of indicator organisms (overnight culture); 0.50 mL of control or G3P-01, or 0.05 mL of positive control agent; and 0.50 mL of S9 mixture or phosphate-buffered saline, for tests with or without metabolic activation, respectively. The tube contents were gently mixed and then poured onto minimal glucose plates. The top agar was allowed to set, and the plates were incubated at 36 to 38 °C for 2 days. After the incubation, revertant colonies were counted manually or using Protocol 3 Colony Counter Software (Synbiosis; Frederick, USA).

The test product was considered positive for mutagenicity if it induced an increase of revertants per plate with increasing concentration. The increases were to be at least 2 times the control background frequency for strains with high spontaneous levels (i.e. TA100) and 3 times for those with low spontaneous levels (i.e. TA98, TA1535, TA1537, and WP2 *uvrA*). These increases were to be observed in at least 2 or more successive concentrations or the response was to be repeatable at a single concentration. The test article was considered to be negative for inducing mutagenicity if it did not induce a response that fulfilled the criteria for a positive response. For each concentration level and condition, the mean revertant count and the standard deviation were determined.

#### In vitro *micronucleus assay*

The test article was characterized in accordance with ISO 17025 standards. G3P-01 (Batch No. 200268) was solubilized in sterile water (Corning) at a target concentration of 20 mg/mL for the concentration range-finding assay and 3 mg/mL for the main micronucleus assay. The lower concentrations were prepared by serial dilutions using dimethyl sulfoxide. A correction factor of 1.75 was used for purity correction during the formulation preparation. The S9 fraction was prepared as described in Section 2.2.1 and used for metabolic activation at 17.5% (v/v) S9.

Human TK6 cells were obtained from Pfizer Global Research and Development (Groton, USA) and were subsequently subcloned. Only cells found to be free of mycoplasma contamination were used. The passage number of the cells was 9 for the concentration range-finding assay and 20 for the main micronucleus assay. TK6 stock cultures were maintained in RPMI 1640 + l-glutamine supplemented with 10% heat inactivated fetal bovine serum and 1% penicillin-streptomycin (Complete Culture Medium; CCM). On the day of dosing, TK6 cells were established in CCM at a cell density of 2.5 to 3.5 × 10^5^ cells/mL. Cultures were incubated at 37 °C ± 1 °C, 5% ± 1% carbon dioxide in flasks.

The TK6 cells were treated with G3P-01, positive controls, or system control in the presence and absence of metabolic activation for short incubations (4 h) and in the absence of metabolic activation for the long incubation (27 h). A harvest time of approximately 27 h was used for the 27-h exposure without metabolic activation, with no recovery period. A harvest time of approximately 44 h was used for the 4-h exposures with and without metabolic activation, with a 40-h recovery period. In the concentration range-finding assay, G3P-01 was tested at concentrations ranging from 3.91 to 2,000 μg/mL for each of the 3 treatment conditions. Based on this preliminary assay, the following concentrations of G3P-01 were selected for the micronucleus assay: 62.5 to 250 μg/mL for the 4-h treatment without metabolic activation; 62.5 to 200 μg/mL for the 27-h treatment without metabolic activation; and 62.5 to 300 μg/mL for the 4-h treatment with metabolic activation. Sterile water (Corning; Manassas, USA) served as a control. The positive controls were as follows: 0.0625 or 0.125 μg/mL mitomycin C (Chemical Abstracts Service [CAS] No. 50-07-7; Sigma-Aldrich, Inc. Lot No. SLBX1864) for the 4-h treatment without metabolic activation; 0.0025 or 0.0030 μg/mL vinblastine sulfate (CAS No. 143-67-9; Sigma-Aldrich, Inc. Lot No. 107M4057V) for the 27-h treatment without metabolic activation; and 4.7 or 11.9 μg/mL cyclophosphamide monohydrate (CAS No. 6055-19-2; Sigma-Aldrich, Inc. Lot No. MKCL2547) for the 4-h treatment with metabolic activation.

Upon harvesting, cultures were resuspended, and an aliquot of each culture was removed for counting via Coulter counter and for micronucleus evaluation via flow cytometry (FACSCanto II, BD BioSciences, Erembodegem, Belgium) and data acquired using FACSDiva Software. Cytotoxicity was assessed using cell count data obtained from Coulter counts and an appropriate calculation of cytotoxicity (i.e. relative population doubling). Cultures with up to 65% cytotoxicity were selected for flow cytometric scoring. From the processed cultures, micronucleus frequencies were analyzed in at least 20,000 nucleated events (approximately 10,000 nucleated events per culture). The highest test article concentration evaluated was (i) the concentration inducing approximately 50 to 60% cytotoxicity; (ii) the lowest concentration where precipitate was observed at the end of the exposure period; or (iii) the limit dose for the assay, whichever was lowest. At least 4 lower concentrations were processed for flow cytometric scoring as applicable.

Cultures for micronucleus evaluation were processed by transferring 1 mL of resuspended culture into 15 mL tubes pre-filled with 3 mL of complete media. The samples were centrifuged at 2,500 × g for 5 min, and the supernatants were aspirated. Pellets were gently re-suspended and submerged on ice for at least 20 min. Reagents (Nucleic Acid Dye A, 1 × Buffer Solution, Complete Lysis Solution 1 and 2) were prepared according to the in vitro MicroFlow kit (Litron Labs; Rochester, USA). After at least 20 min on ice, pellets were triturated with 300 μL Nucleic Acid Dye A. With the tubes submerged in ice, a fluorescent light was placed above the tubes for 30 ± 5 min. The light was removed, and 3 mL of cold 1 × Buffer Solution was added. Samples were centrifuged at 2,500× g for 5 min, supernatants were aspirated, and pellets were tapped to re-suspend. To lyse cells, 400 μL of Complete Lysis Solution 1 was added, and samples were incubated for 60 ± 10 min at ambient temperature, protected from light, followed by the addition of 400 μL Complete Lysis Solution 2 containing fluorescent beads. A FACSCanto II with FACSDiva Software was used for data acquisition and analysis. For statistical analyses, a z-test was applied to detect statistically significant differences in the percentage of induced micronuclei between control and treatment groups.

The test article was considered positive for inducing micronuclei if a significant increase (z’ ≥ 0.6) in the percentage of cells with micronuclei was observed at 1 or more dose levels. If a significant increase was observed at 1 or more dose levels, a dose-response would be expected, defined as a statistically significant Cochran-Armitage test (*P* ≤ 0.05). The trend test can only be performed if pairwise comparisons demonstrate a statistically significant response. Additionally, in the event of a significant increase, at least 1 concentration would be expected to be associated with an increase above the upper bounds of the vehicle-control-treated historical control range.^1^ The test product was considered negative for inducing micronuclei if the positive response criteria were not met.

#### 13-week repeat-dose toxicity study

This study was conducted in compliance with all applicable sections of the Public Health Service Policy on Humane Care and Use of Laboratory Animals from the Office of Laboratory Animal Welfare,^39^ and the Guide for the Care and Use of Laboratory Animals from the National Research Council.^36^ The Protocol and any amendments or procedures involving the care or use of animals were reviewed and approved by the Testing Facility Institutional Animal Care and Use Committee before the initiation of such procedures. The Protocol and any amendment(s) or procedures involving the care and use of animals in this study were reviewed and approved by Charles River Ashland Institutional Animal Care and Use Committee (IACUC) before conduct. During the study, the care and use of animals were conducted with guidance from the guidelines of the USA National Research Council.

##### Preparation of formulations

G3P-01 was added to Rodent Diet AIN-93G (TestDiet®, Richmond, Indiana, USA) on a weight/weight basis, corrected for purity, to obtain dose levels of 0, 9,000, 18,000, and 36,000 ppm. The formulations were prepared at least weekly and maintained at 18 to 24 °C. Samples were collected from different strata of the preparation vessel to analyze for homogeneity. For concentration analysis, samples were collected from all groups from the middle stratum of the preparation container for the first and last formulation preparation. The samples were stored at −20 °C until analysis. For stability analysis, dietary mixtures were prepared at 9,000 ppm and 36,000 ppm and analyzed after 15 days of storage at room temperature and after 9 months of storage at −18 °C.

##### Analysis of G3P-01 in the feed

Analyses of feed were performed at Eurofins Food Testing Laboratory BV (Heerenveen, The Netherlands) using a validated analytical procedure to determine the concentrations of the monosaccharide sugars in the soluble fiber fraction of the diets. The concentrations of G3P-01 in the diet were indirectly verified by the quantification of hydrolyzed monosaccharide sugar units of the active pectin ingredient in the test substance. The concentrations of monosaccharide units in the basal diet were analyzed to determine the background levels of monosaccharides. Galactose, arabinose, galacturonic acid (represented as uronic acid), rhamnose, fucose, xylose, mannose, and glucose were analyzed in both the soluble and insoluble fraction of the samples. The concentration of the total soluble non-starch fraction, including non-starch polysaccharides (NSPs), was low in basal diet and increased in a concentration-dependent manner in the test diets. Thus, NSPs were used to calculate the amount of G3P-01 added to the test diets. To quantify the NSPs, samples were defatted twice with petroleum ether and subsequently treated with α-amylase and amyloglucosidase according to AOAC 994.13^40^ to hydrolyze the starch. Samples were centrifuged, and the total soluble NSP fraction was treated with 80% ethanol to precipitate the soluble fibers/NSPs. The obtained insoluble fraction and precipitated soluble fiber or NSP fractions were hydrolyzed with 1 M H_2_SO_4_ at 100 °C for 120 min (adapted from Englyst and Cummings,^41^ with omission of the first hydrolysis step). The released monosaccharides were separated and quantified using high-performance anion exchange chromatography with pulsed amperometric detection (HPAEC-PAD, Dionex, Thermo Fisher Scientific, Sunnyvale, CA, USA). The total uronic acid content in the hydrolysates was determined by the phenylphenol colorimetric method described by De Ruiter et al.^42^

##### Animals

For the 13-week study, 45 male and 45 female Crl:CD Sprague-Dawley rats were obtained from Charles River Laboratories (Raleigh, USA). The animals were approximately 7 weeks old. At the start of dosing, the females weighed between 137 and 200 g and the males weighed between 202 and 263 g. Animals judged to be in good health were placed in acclimation for at least 12 days. The animals (n = 10/sex/group) were assigned to dose level groups for the 13-week toxicity study. For the recovery study, 5 female and 5 male rats were assigned to groups receiving 0 or 36,000 ppm G3P-01 in the diet for the 13-week study, with a subsequent 28-day recovery period. Animals were assigned to groups by a stratified randomization scheme designed to achieve similar group mean body weights. Males and females were randomized separately. Individual body weights at randomization were within ±20% of the mean for each sex. Following the group randomization procedure, the animals were randomly assigned to replicates for functional observational (FOB) assessment using an appropriate computer program. Each dose group and sex were approximately equally represented in each study replicate to avoid introduction of bias into the recorded observations related to these variables.

Two to three animals of the same sex and same dosing group were housed together. Animals were held in solid-bottom cages containing appropriate bedding material (Bed-O-Cobs®, the Andersons, USA, or other suitable material). The housing set-up was as specified in the *Guide for the Care and Use of Laboratory Animals.*^36^ For enrichment, animals were provided items such as treats, a gnawing device, and/or nesting material, except when interrupted by study procedures or activities. Animals were held in a controlled environment with a daily temperature of 20 to 26 °C, humidity of 30 to 70%, and 12-h light and dark cycles. Feed was provided ad libitum, except during designated fasting periods. Tap water (municipal tap water, automatic watering system) was provided ad libitum.

##### Experimental design

G3P-01 was administered continuously in the diet for at least 91 consecutive days (13 weeks). The dose levels were based on previously conducted palatability and oral gavage dose range–finding studies in Sprague-Dawley rats. A dose range-finding study was previously conducted using dose levels of 1, 10, 100, and 1,000 mg/kg body weight in a dose-escalating manner phase, and at a single dose level of 2,000 mg/kg body weight/day for a 30-day repeat-dose phase. No adverse effects were reported during either phase of the study, and the dose level of 2,000 mg/kg body weight/day was considered to be well tolerated via oral gavage. To consider the intended route of exposure in humans, a 14-day dietary oral palatability and range-finding study was conducted with G3P-01 at dose levels of 9,000, 18,000, and 36,000 ppm in the AIN-93G diet. The mid- and low-dose concentrations in the palatability study were selected to be one-half and one-quarter of the high-dose, respectively, in anticipation of graded responses to the test substance. All dose levels were considered tolerable during the palatability study. Therefore, the same dose levels of G3P-01 at 9,000, 18,000 and 36,000 ppm were applied to the main 13-week repeat-dose toxicity study.

##### Observations and measurements

Cage-side observations, including mortality, were assessed at least once daily. Detailed clinical observations and individual body weights were recorded within 4 days of receiving the animals, as well as on the day of randomization and weekly during the study period. Food consumption was quantitatively measured once weekly, beginning on Day 1. Intake of G3P-01 in mg/kg body weight/day was calculated for each cage using the number of day interval, the test substance concentration, and the body and feed weight at the start and end of the interval. Data were presented as a cage mean for each period of food consumption and as an overall achieved dose, which was the arithmetic mean of all calculations during the treatment period for each group. Food utilization (body weight gained as a percent of food consumed) was calculated per cage using the number of days of the measurement interval and body and food weight at the start and end of the interval. Determination of estrous cycles was performed on female rats on the day of scheduled necropsy.

Ophthalmic examinations, neurobehavioral assessments, and motor activity evaluations were performed once during the pretreatment period and near the end of the dosing period (Day 91; Week 13). Ophthalmic examinations were conducted by a board-certified veterinary ophthalmologist using an indirect ophthalmoscope and slit lamp biomicroscope. Prior to examination, animals were treated with a mydriatic agent. Neurobehavioral assessment included observations of FOB parameters. Motor activity evaluated total and ambulatory activity counts obtained during a 60-min test session.

Hematology, coagulation, clinical chemistry (after 8 h of fasting), and urinalysis samples were taken on the day of scheduled necropsy. Blood samples were analyzed for red blood cell count, hemoglobin concentration, hematocrit, mean corpuscular volume, red blood cell distribution width, mean corpuscular hemoglobin concentration, mean corpuscular hemoglobin, reticulocyte count (absolute), platelet count, white blood cell count, neutrophil count (absolute), lymphocyte count (absolute), monocyte count (absolute), eosinophil count (absolute), basophil count (absolute), large unstained cells (absolute), and other cells, as appropriate. Blood samples were processed for plasma, and the plasma was analyzed for activated partial thromboplastin time (APTT), fibrinogen, prothrombin time, and sample quality (degree of hemolysis, lipemia, and icterus). Blood serum was analyzed for alanine aminotransferase (ALT), aspartate aminotransferase (AST), alkaline phosphatase (ALP), *gamma*-glutamyltransferase (GGT), creatine kinase, total bilirubin, urea nitrogen, creatinine, calcium, phosphorus, total protein, albumin, globulin (calculated), albumin/globulin ratio, glucose, cholesterol, low-density lipoprotein (LDL) cholesterol, high-density lipoprotein (HDL) cholesterol, triglycerides, sodium, potassium, chloride, and sample quality.

Urine samples were analyzed for color, appearance/clarity, specific gravity, pH, volume, protein, glucose, bilirubin, ketones, blood, and urobilinogen.

Blood samples for thyroid hormone analyses were taken the day prior to scheduled necropsy using venipuncture from a jugular vein. Samples were allowed to clot at ambient temperature before centrifugation. Resultant serum was separated and used for triiodothyronine (T3), T4 (thyroxine), and thyroid-stimulating hormone (TSH) analysis. For total T3 and T4 analysis, hormone samples were analyzed using validated ultra-high-performance liquid chromatography with dual mass spectroscopy assays. Analysis of serum samples to determine TSH concentrations was conducted using a validated Luminex Bead Based assay.

The animals were euthanized after weighing on Day 93 (males) or Day 94 (females) using carbon dioxide inhalation. Recovery study animals were euthanized on Day 121 (males) or Day 122 (females). Animals were fasted overnight before the scheduled necropsies. Organs were weighed and organ-to-body weight ratios (using the terminal body weight) and organ to brain weight ratios were calculated Animals from the main and recovery studies were subjected to a complete necropsy examination, which included evaluation of the carcass; all external surfaces and orifices; cranial cavity and external surfaces of the brain; and thoracic, abdominal, and pelvic cavities with their associated organs and tissues.

Tissue samples of all essential organs were collected from all animals and preserved in 10% neutral buffered formalin. Tissues from animals in the control and high-dose groups at the terminal necropsy were embedded in paraffin, sectioned, mounted on glass slides, and stained with hematoxylin and eosin and examined microscopically. In addition, gross lesions (macroscopic abnormalities) were assessed in all animals in the low- and mid-dose groups at the terminal necropsy and all animals at the recovery necropsy.

##### Statistical analysis

Levene’s test was used to assess the homogeneity of group variances in body weight, body weight gains, food consumption, neurobehavioral assessment data (FOB data), hematology variables, coagulation variables, clinical chemistry variables, urinalysis variables, organ weights, organ weight relative to body weight, organ weight relative to brain weight, and TSH. The groups were compared using an overall one-way analysis of variance (ANOVA) F-test if Levene’s test was not significant or the Kruskal-Wallis test if it was significant. If the overall F-test or Kruskal-Wallis test was found to be significant, then pairwise comparisons were conducted using Dunnett’s or Dunn’s test, respectively. For food utilization, the motor activity data and T3 and T4 variables of groups were compared using an overall one-way ANOVA F-test. If the overall F-test was found to be significant, then pairwise comparisons were conducted using Dunnett’s test. All statistical tests were conducted at the 5% significance level. All pairwise comparisons (Group 1 vs. Group 2, 3, or 4) were conducted using two-sided tests and are reported at the 1 and 5% levels, unless otherwise noted. Analyses excluded any group with less than 3 observations.

## Results

### Bacterial reverse mutation test

In the concentration range-finding study, no cytotoxicity (i.e. reduction in the background lawn and/or mean number of revertant colonies) was observed in strain TA100 or WP2 *uvr*A, with or without metabolic activation. In other strains and concentrations, the criteria for a positive response were not met. Precipitates were observed at ≥1,000 μg/plate in both strains, with and without metabolic activation.

In the main mutagenicity assay, cytotoxicity was not observed in any strain, with or without metabolic activation. The number of mean revertant colonies did not increase significantly in any treatment group compared to the negative control, and the criteria for a negative response were met for all strains with and without metabolic activation (Table 2). Precipitates were observed at concentrations ≥500 μg/plate in all strains, with and without metabolic activation. The mean revertant colonies in all positive control groups increased significantly more than 2-fold compared to the negative controls.

**Table 2:** Results of the bacterial reverse mutation test on G3P-01.

Treatment Group	Concentration (μg/plate)	Revertant colonies per plate (mean ± SD)				
		*Salmonella typhimurium*				*Escherichia coli*
		TA98	TA100	TA1535	TA1537	WP2 *uvrA*
Without Metabolic Activation						
Negative control	0.5 mL/plate	16 ± 5	128 ± 9	14 ± 5	8 ± 5	50 ± 6
G3P-01	25	12 ± 3	142 ± 7	14 ± 3	5 ± 3	50 ± 25
	50	15 ± 2	136 ± 8	8 ± 3	10 ± 5	62 ± 14
	100	16 ± 2	141 ± 16	9 ± 3	5 ± 4	56 ± 17
	250	14 ± 2	166 ± 16	13 ± 8	10 ± 7	42 ± 10
	500^a^	17 ± 10	163 ± 4	12 ± 6	11 ± 1	50 ± 12
	1,000^a^	13 ± 5	141 ± 28	13 ± 2	9 ± 3	41 ± 9
	2,500^a^	16 ± 6	181 ± 7	20 ± 6	10 ± 1	31 ± 10
Positive controls						
2-Nitrofluorene	2.5	642 ± 41^b^	NA	NA	NA	NA
Sodium azide	1.0	NA	361 ± 16^b^	489 ± 47^b^	NA	NA
ICR-191 acridine	0.5	NA	NA	NA	117 ± 46^b^	NA
4-Nitroquinoline-N-oxide	2.0	NA	NA	NA	NA	906 ± 78^b^
With Metabolic Activation						
Negative control	0.5 mL/plate	11 ± 3	127 ± 23	12 ± 2	6 ± 0	50 ± 26
G3P-01	25	15 ± 3	135 ± 18	11 ± 4	9 ± 2	55 ± 15
	50	15 ± 5	166 ± 29	11 ± 5	12 ± 10	48 ± 29
	100	21 ± 3	153 ± 19	14 ± 1	4 ± 2	30 ± 3
	250	15 ± 6	150 ± 28	13 ± 4	10 ± 8	32 ± 2
	500^a^	12 ± 7	151 ± 35	12 ± 1	9 ± 6	42 ± 23
	1,000^a^	14 ± 3	151 ± 10	18 ± 4	5 ± 4	39 ± 12
	2,500^a^	20 ± 2	174 ± 8	20 ± 2	10 ± 4	38 ± 8
Positive controls						
2-Aminoanthracene	2.5	2,285 ± 137^b^	1,587 ± 643^b^	164 ± 54^b^	160 ± 104^b^	NA
2-Aminoanthracene	10	NA	NA	NA	NA	298 ± 87^b^

G3P-01 = rhamnogalacturonan-I-enriched pectin extract; NA = not applicable; SD = standard deviation.

^a^Precipitates present.

^b^Criteria for a positive response met; criteria for a positive response: 2-fold (TA 100) or 3-fold (TA 98, TA 1535, TA 1537, and WP2 *uvrA*) increase in revertant colonies relative to negative controls.

### In vitro micronucleus assay

In the micronucleus assay, precipitates were observed in all G3P-01 treatment groups from 179 μg/mL and above. The concentrations selected for micronucleus evaluation in the 4-h treatment without metabolic activation were based on precipitates and were as follows (with percent cytotoxicity): 100 μg/mL (3%), 118 μg/mL (14%), 152 μg/mL (13%), 165 μg/mL (18%), and 179 μg/mL (17%, lowest precipitating dose). The concentrations selected for micronucleus evaluation in the 27-h treatment without metabolic activation were based on cytotoxicity and precipitates and were as follows (with percent cytotoxicity): 62.5 μg/mL (9%), 100 μg/mL (15%), 128 μg/mL (27%), 165 μg/mL (44%), and 179 μg/mL (59%, lowest precipitating dose). The concentrations selected for micronucleus evaluation in the 4-h treatment with metabolic activation were based on precipitation and were as follows (with percent cytotoxicity): 62.5 μg/mL (0%), 128 μg/mL (7%), 152 μg/mL (11%), and 179 μg/mL (15%, lowest precipitating dose). Micronuclei were evaluated in at least 10,000 cells per culture. No statistically significant increases in the percent of micronucleated cells were observed between G3P-01-treated cultures and the concurrent control under any assay condition (Table 3). Data for the system control and positive controls were comparable to the relevant historical control data.

**Table 3:** Results of in vitro micronucleus assay.

Treatment Group	Concentration (μg/mL)	Toxicity^a^ Mean %	% Micronuclei^b^^,^^c^	z’^d^
4-Hour Treatment Without Metabolic Activation				
Sterile water	10 (%)	0	0.65	NA
G3P-01	100	3	0.61	<0
	118	14	0.84	<0
	152	13	0.80	<0
	165	18	0.68	<0
	179^e^	17	0.90	<0
Mitomycin C	0.0625	17	4.41	^*^0.84
	0.125	45	12.12	^*^0.92
4-Hour Treatment With Metabolic Activation				
Sterile water	10 (%)	0	0.49	NA
G3P-01	62.5	0	0.46	<0
	128	7	0.43	<0
	152	11	0.53	<0
	179^e^	15	0.46	<0
Cyclophosphamide	4.7	24	4.80	^*^0.86
	11.9	48	9.65	^*^0.92
27-Hour Treatment Without Metabolic Activation				
Sterile water	10 (%)	0	0.61	NA
G3P-01	62.5	9	0.67	<0
	100	15	0.78	<0
	128	27	1.56	0.55
	165	44	1.56	0.55
	179^e^	59	1.42	0.49
Vinblastine sulfate	0.0025	25	4.52	^*^0.85
	0.003	35	4.85	^*^0.85

G3P-01 = rhamnogalacturonan-I-enriched pectin extract; NA = not applicable; RPD = relative population doubling.

1. Absence of metabolic activation at 4/40 h

a) Vehicle control: % of cells with micronucleus = 0.44 ± 0.24

b) Mitomycin:

i. Concentration: 0.0625 μg/mL, % of cells with micronucleus = 2.31 ± 1.15

ii. Concentration; 0.125 μg/mL, % of cells with micronucleus = 6.53 ± 2.9

2. Absence of metabolic activation at 27 h

a) Vehicle control: % of cells with micronucleus = 0.25 ± 0.19

b) Vinblastine sulfate:

i. Concentration: 0.0015 μg/mL, % of cells with micronucleus = 4.66 ± 3.47

ii. Concentration: 0.0025 μg/mL, % of cells with micronucleus = 4.72 ± 3.62

iii. Concentration: 0.0030 μg/mL, % of cells with micronucleus = 4.54 ± 2.49

3. Presence of metabolic activation at 4/40 h

a) Vehicle control: 0.46 ± 0.27

b) Cyclophosphamide

i. Concentration: 4.7 μg/mL, % of cells with micronucleus = 2.19 ± 1.67

ii. Concentration; 11.9 μg/mL, % of cells with micronucleus = 7.24 4.5

^a^Toxicity = (100-RPD), where RPD = (the number of population doublings in the treated cultures/the number of population doublings in the negative control cultures) × 100; if RPD is ≤0, it is reported as 0.

^b^The total number of micronuclei/the total number of nuclei events analyzed.

^c^Historical data for micronucleus is as follows:

^d^^*^z’ > 0.6 = significant.

^e^Precipitates noted at end of treatment.

### 13-week repeat-dose toxicity study

#### G3P-01 dose and stability analyses

Arabinose, galactose, uronic acid, and NSPs were analyzed as surrogate markers for G3P-01 in the dietary formulation samples for the low- and high-dose groups. The amount of G3P-01 added to the diet for each concentration and the calculated amounts of G3P-01 using the carbohydrate markers were generally similar; for all markers and at both levels of G3P-01, the calculated amounts of G3P using these markers are close to the expected amount of G3P-01 in the rat diet samples. Data based on galactose and NSP (excluding samples frozen for 9 months) were closest to the expected values of G3P-01 in the diet (up to about 20% higher than the expected value), thus confirming the stability of G3P-01 under the conditions of the study.

#### Clinical and neurobehavioral observations

All animals survived until the scheduled necropsies. Test substance-related clinical observations included yellow feces on Days 22 and 85 for the low-dose group and between Days 8 and 106 for the mid- and high-dose groups. Pale feces were observed in control group animals between Days 29 and 43. No adverse findings were observed in the ophthalmic evaluations. No significant differences were observed in the neurobehavioral assessments for activity, excitability, and autonomic, neuromuscular, physiological, or sensorimotor evaluations.

#### Body weight, feed consumption, and organ weights

**Fig. 2:**
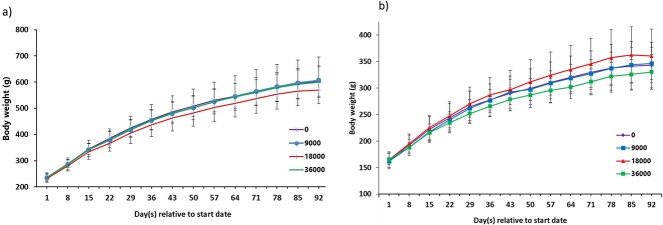
Average body weight of a) male and b) female Sprague-Dawley rats administered G3P 01 in the diet for 13 weeks.

No test-related, dose-response effects were observed in relation to food consumption. The average consumption of G3P-01 from the 9,000, 18,000, and 36,000 ppm treatment groups was 455, 894, and 1,898 mg/kg body weight for males and 549, 1,192, and 2,361 mg/kg body weight/day for females, respectively. No test substance-related effects on body weight were determined (Fig. 2). During the recovery period, the mean body weight for high-dose females was 16.5% lower than the mean body weight of the control group.^2^ However, individual body weights in this treatment group were not significantly different from the control group.

#### Organ weights

Significant (*P* < 0.01) increases were reported in cecal weights (with cecal contents) in male rats treated with 36,000 ppm G3P-01. There was an increase in empty cecal weight in male rats that consumed 36,000 ppm G3P-01 and a significant increase in the empty cecal weight in female rats that consumed 18,000 ppm (*P* < 0.05) and 36,000 ppm (*P* < 0.01) G3P-01 at the end of the study period (Table 4). No corresponding microscopic changes in the cecum were observed. Significant reduction was observed in relative organ weight for liver in the mid-dose (*P* < 0.01) and high-dose (*P* < 0.05) males. No other significant differences were observed for any absolute or relative organ weights.

**Table 4:** Absolute and relative organ weights for Sprague-Dawley rats administered G3P-01 in the diet for 13 weeks.

Parameter (mean ± SD)	Dose group (ppm)							
	Males				Females			
	0 (control)	9,000	18,000	36,000	0 (control)	9,000	18,000	36,000
Terminal body weight (g)	581.2 ± 67.1	589.2 ± 86.6	552.3 ± 63.8	569.5 ± 60.7	317.9 ± 47.8	328.8 ± 30.7	344.4 ± 50.4	325.2 ± 31.2
**Absolute organ weight (g)**								
Brain	2.21 ± 0.11	2.17 ± 0.11	2.17 ± 0.11	2.18 ± 0.07	2.01 ± 0.10	2.00 ± 0.07	2.03 ± 0.10	2.01 ± 0.06
Adrenal gland	0.059 ± 0.010	0.058 ± 0.011	0.056 ± 0.011	0.055 ± 0.008	0.069 ± 0.014	0.070 ± 0.008	0.078 ± 0.013	0.073 ± 0.012
Pituitary gland	0.016 ± 0.002	0.013 ± 0.002	0.015 ± 0.003	0.015 ± 0.002	0.020 ± 0.003	0.021 ± 0.004	0.019 ± 0.003	0.019 ± 0.004
Thyroid/parathyroid	0.027 ± 0.005	0.028 ± 0.005	0.027 ± 0.003	0.029 ± 0.003	0.021 ± 0.003	0.020 ± 0.001	0.021 ± 0.003	0.020 ± 0.003
Heart	1.79 ± 0.24	1.72 ± 0.22	1.64 ± 0.18	1.77 ± 0.28	1.10 ± 0.17	1.10 ± 0.09	1.13 ± 0.11	1.10 ± 0.08
Kidney	3.48 ± 0.45	3.31 ± 0.39	3.19 ± 0.41	3.41 ± 0.37	2.00 ± 0.27	1.92 ± 0.15	1.99 ± 0.20	2.03 ± 0.25
Cecum, full	2.48 ± 0.40	2.78 ± 0.64	2.69 ± 0.40	3.58 ± 1.04^a^	1.73 ± 0.40	1.66 ± 0.32	1.97 ± 0.58	2.16 ± 0.54
Cecum, empty	1.11 ± 0.17	1.15 ± 0.14	1.21 ± 0.13	1.39 ± 0.20^***^	0.83 ± 0.18	0.83 ± 0.12	1.04 ± 0.20^b^	1.10 ± 0.18^***^
Liver	15.54 ± 2.76	15.65 ± 2.72	13.09 ± 1.57	13.87 ± 2.01	9.41 ± 0.93	9.19 ± 0.75	9.42 ± 1.06	9.43 ± 0.87
Spleen	0.90 ± 0.23	0.87 ± 0.16	0.79 ± 0.15	0.78 ± 0.13	0.53 ± 0.07	0.52 ± 0.04	0.52 ± 0.09	0.50 ± 0.05
Thymus	0.30 ± 0.07	0.24 ± 0.06	0.31 ± 0.08	0.29 ± 0.10	0.33 ± 0.21	0.28 ± 0.08	0.27 ± 0.06	0.27 ± 0.07
Epididymis	1.27 ± 0.15	1.31 ± 0.12	1.32 ± 0.13	1.36 ± 0.12	NA	NA	NA	NA
Prostate/seminal vesicle	3.83 ± 0.32	3.56 ± 0.38	3.60 ± 0.55	3.76 ± 0.52	NA	NA	NA	NA
Testis	3.57 ± 0.27	3.58 ± 0.29	3.43 ± 0.19	3.79 ± 0.32	NA	NA	NA	NA
Uterus with cervix	NA	NA	NA	NA	0.65 ± 0.20	0.52 ± 0.10	0.66 ± 0.17	0.68 ± 0.22
Ovary/oviduct	NA	NA	NA	NA	0.12 ± 0.03	0.13 ± 0.02	0.14 ± 0.03	0.13 ± 0.02
**Relative organ weight (%)**								
Brain	0.38 ± 0.03	0.38 ± 0.06	0.40 ± 0.06	0.39 ± 0.05	0.64 ± 0.09	0.61 ± 0.05	0.59 ± 0.08	0.62 ± 0.05
Adrenal gland	0.010 ± 0.002	0.010 ± 0.002	0.010 ± 0.001	0.010 ± 0.001	0.022 ± 0.005	0.021 ± 0.002	0.023 ± 0.005	0.023 ± 0.004
Pituitary gland	0.0028 ± 0.0004	0.0023 ± 0.0004	0.0027 ± 0.0004	0.0026 ± 0.0003	0.006 ± 0.001	0.006 ± 0.001	0.006 ± 0.001	0.006 ± 0.001
Thyroid/parathyroid	0.0046 ± 0.0006	0.0047 ± 0.0008	0.0050 ± 0.0007	0.0051 ± 0.0006	0.007 ± 0.001	0.006 ± 0.001	0.006 ± 0.001	0.006 ± 0.001
Heart	0.31 ± 0.03	0.29 ± 0.03	0.30 ± 0.02	0.31 ± 0.03	0.35 ± 0.03	0.34 ± 0.04	0.32 ± 0.02	0.34 ± 0.02
Kidneys	0.60 ± 0.07	0.57 ± 0.06	0.58 ± 0.06	0.60 ± 0.06	0.64 ± 0.09	0.59 ± 0.05	0.57 ± 0.08	0.63 ± 0.07
Cecum, empty	0.19 ± 0.02	0.20 ± 0.03	0.22 ± 0.03	0.25 ± 0.03^***^	0.26 ± 0.06	0.25 ± 0.02	0.31 ± 0.08	0.34 ± 0.06^b^
Liver	2.66 ± 0.22	2.65 ± 0.19	2.37 ± 0.17^***^	2.43 ± 0.20^b^	2.99 ± 0.33	2.80 ± 0.18	2.70 ± 0.32	2.92 ± 0.31
**Relative organ weight (%)—Continued**								
Spleen	0.153 ± 0.024	0.147 ± 0.016	0.143 ± 0.020	0.137 ± 0.019	0.17 ± 0.02	0.16 ± 0.02	0.15 ± 0.03	0.15 ± 0.02
Thymus	0.051 ± 0.014	0.041 ± 0.006	0.056 ± 0.012	0.051 ± 0.017	0.11 ± 0.09	0.086 ± 0.023	0.079 ± 0.020	0.085 ± 0.022
Epididymis	0.22 ± 0.04	0.23 ± 0.03	0.24 ± 0.03	0.24 ± 0.03	NA	NA	NA	NA
Prostate/seminal vesicle	0.67 ± 0.09	0.62 ± 0.13	0.65 ± 0.10	0.66 ± 0.10	NA	NA	NA	NA
Testis	0.62 ± 0.06	0.62 ± 0.11	0.63 ± 0.09	0.67 ± 0.09	NA	NA	NA	NA
Uterus with cervix	NA	NA	NA	NA	0.20 ± 0.04	0.16 ± 0.03	0.19 ± 0.07	0.21 ± 0.06
Ovary/oviduct	NA	NA	NA	NA	0.04 ± 0.01	0.04 ± 0.01	0.04 ± 0.01	0.04 ± 0.01

G3P-01 = rhamnogalacturonan-I-enriched pectin extract; NA = not applicable; ppm = parts per million; SD = standard deviation.

^a^Significantly different from control (Kruskal-Wallis & Dunn, *P* ≤ 0.01).

^b^Significantly different from control (Anova & Dunnett, *P* ≤ 0.05; ^***^P ≤ 0.01).

#### Motor activity

No test substance-related changes were observed for motor activity patterns (total and ambulatory activity counts). No statistically significant changes in motor activity were noted in males when compared to the control group after 13 weeks of oral exposure to G3P-01. Statistically significant lower mean total and ambulatory counts were noted in the low-dose females when compared to the control group. These changes were not observed at the higher dose levels. In addition, the total and ambulatory counts in the low-dose females, measured before the administration of the test substance during pretreatment period, were 15 to 18% lower when compared to the control group females. Thus, there were no remarkable shifts in the patterns of habituation in any of the test substance–treated groups when the animals were evaluated in Week 13.

#### Clinical chemistry and urinalysis

Some test substance-related changes in clinical chemistry parameters were reported at the end of the study period. For example, in male rats of the 18,000 ppm G3P-01 group, there was a significant (*P* < 0.05) reduction in serum cholesterol level, as well as a highly significant (*P* < 0.01) reduction in cholesterol level in the 36,000 ppm G3P-01 group in comparison with the control group (Table 5). This decrease in mean lower cholesterol (0.77-fold) and LDL (0.78-fold) persisted until the end of the recovery period in the 36,000 ppm G3P-01 group males. Although there was a reduction in total cholesterol level in the G3P-01 groups in female rats, this did not achieve statistical significance. Further, there was a 0.67-fold statistically significant (*P* < 0.05) reduction in LDL in males of the 36,000 ppm G3P-01 group in comparison with the control group. However, this effect was not observed in female rats at any of the dose levels. Plasma levels of HDL were not affected in any of the treatment groups, male or female. Plasma protein levels were not affected by daily ingestion of G3P-01, except for the statistically significant (*P* < 0.05) reduction in globulin and a significant increase in the albumin-globulin ratio in male rats that received 36,000 ppm G3P-01 in the diet. The markers of liver functions, such as ALT, AST, ALP, and GGT, were not adversely affected by daily exposure to G3P-01 at any dose level. In male rats exposed to the test article, there were reductions, although not statistically significant, in AST and ALT. A similar observation was made for markers of renal function, wherein no significant effect was reported for creatinine, urea nitrogen, and creatine kinase in any of the treatment groups. Although there was an increase in the level of creatine kinase in female rats of the 36,000 ppm G3P-01 group, this increase was not statistically significant. Other biochemical parameters measured, such as urinalysis and electrolytes, among others, remained unaffected after 13 weeks of oral exposure to the test article. No other significant changes in clinical chemistry or urinalysis parameters were observed.

**Table 5:** Clinical chemistry and urinalysis results for Sprague-Dawley rats administered G3P-01 in the diet for 13 weeks.

Parameter (mean ± SD)	Dose group (ppm)							
	Males				Females			
	0 (control)	9,000	18,000	36,000	0 (control)	9,000	18,000	36,000
**Clinical chemistry**								
AST (U/L)	157.3 ± 67.2	127.1 ± 36.7	122.1 ± 22.0	119.2 ± 16.8	100.9 ± 17.4	101.2 ± 21.8	117.7 ± 22.5	119.1 ± 36.9
ALT (U/L)	58.1 ± 48.7	42.6 ± 28.1	29.8 ± 6.4	28.4 ± 3.6	20.8 ± 4.3	18.9 ± 3.0	21.3 ± 2.0	27.9 ± 15.3
ALP (U/L)	79.6 ± 14.8	92.4 ± 24.8	103.3 ± 12.6	93.5 ± 23.0	37.4 ± 7.9	41.8 ± 9.6	46.3 ± 14.8	53.1 ± 14.0
GGT (U/L)	0.0 ± 0.0	0.0 ± 0.0	0.0 ± 0.0	0.0 ± 0.0	0.1 ± 0.3	0.1 ± 0.3	0.4 ± 0.8	0.0 ± 0.0
Creatinine kinase (U/L)	766.7 ± 291.2	716.8 ± 310.7	750.3 ± 284.2	697.2 ± 262.5	768.6 ± 446.8	623.9 ± 322.6	719.8 ± 219.2	1371.7 ± 1789.1
Total bilirubin (mg/dL)	0.041 ± 0.022	0.043 ± 0.022	0.037 ± 0.024	0.031 ± 0.020	0.070 ± 0.010	0.070 ± 0.022	0.069 ± 0.030	0.074 ± 0.013
Urea nitrogen (mg/dL)	10.7 ± 1.2	11.6 ± 1.2	12.4 ± 1.4	11.4 ± 1.6	12.8 ± 2.9	12.4 ± 2.1	12.7 ± 2.4	13.0 ± 2.7
Creatinine (mg/dL)	0.31 ± 0.03	0.31 ± 0.04	0.31 ± 0.04	0.28 ± 0.03	0.39 ± 0.04	0.38 ± 0.03	0.40 ± 0.05	0.40 ± 0.05
Glucose (mg/dL)	103.8 ± 11.0	107.4 ± 9.0	102.6 ± 6.4	104.0 ± 8.5	88.2 ± 10.6	95.1 ± 7.3	92.1 ± 9.9	94.4 ± 10.4
Total cholesterol (mg/dL)	93.8 ± 15.9	96.1 ± 25.6	69.4 ± 21.0^a^	67.6 ± 8.2^****^	102.2 ± 18.8	89.8 ± 22.0	97.6 ± 16.9	91.2 ± 14.7
HDL (mg/dL)	18.9 ± 2.9	20.4 ± 4.3	15.2 ± 3.8	15.9 ± 1.4	27.0 ± 5.4	24.2 ± 5.1	25.6 ± 3.6	26.3 ± 3.1
LDL (mg/dL)	18.9 ± 4.7	17.9 ± 6.1	14.1 ± 5.2	12.7 ± 3.6^b^	11.6 ± 3.1	10.3 ± 3.6	12.0 ± 3.5	10.1 ± 2.9
Triglycerides (mg/dL)	98.1 ± 43.7	163.3 ± 90.7	100.3 ± 47.3	91.3 ± 52.7	66.9 ± 50.8	60.1 ± 18.3	57.1 ± 19.8	55.2 ± 31.6
Total protein (g/dL)	6.68 ± 0.21	6.72 ± 0.32	6.49 ± 0.35	6.47 ± 0.25	8.02 ± 0.44	7.89 ± 0.47	7.85 ± 0.53	7.97 ± 0.62
Albumin (g/dL)	4.36 ± 0.14	4.37 ± 0.19	4.26 ± 0.20	4.32 ± 0.18	5.36 ± 0.34	5.26 ± 0.42	5.15 ± 0.28	5.28 ± 0.44
Globulin (g/dL)	2.32 ± 0.12	2.35 ± 0.16	2.23 ± 0.17	2.15 ± 0.10^b^	2.67 ± 0.25	2.63 ± 0.16	2.70 ± 0.35	2.69 ± 0.25
A/G ratio	1.90 ± 0.09	1.87 ± 0.11	1.93 ± 0.09	2.03 ± 0.05^**^	2.02 ± 0.24	2.00 ± 0.17	1.92 ± 0.22	1.97 ± 0.17
Calcium (mg/dL)	10.67 ± 0.19	10.73 ± 0.26	10.54 ± 0.25	10.47 ± 0.18	11.63 ± 0.37	11.50 ± 0.47	11.55 ± 0.37	11.68 ± 0.43
Inorganic phosphorus (mg/dL)	7.51 ± 0.33	7.27 ± 0.40	7.49 ± 0.36	7.58 ± 0.42	6.96 ± 0.40	7.14 ± 0.45	6.97 ± 0.33	7.46 ± 0.84
**Clinical chemistry—Continued**								
Sodium (mEq/L)	144.5 ± 1.3	144.5 ± 0.8	144.6 ± 0.5	144.8 ± 0.9	143.2 ± 1.0	142.5 ± 1.0	142.9 ± 1.0	143.6 ± 1.6
Potassium (mEq/L)	5.77 ± 0.26	5.50 ± 0.22	5.59 ± 0.30	5.60 ± 0.24	5.26 ± 0.32	5.43 ± 0.39	5.55 ± 0.33	5.50 ± 0.36
Chloride (mEq/L)	104.2 ± 1.1	104.0 ± 0.8	104.6 ± 0.8	105.1 ± 0.9	103.6 ± 1.1	103.6 ± 1.3	103.5 ± 1.0	103.9 ± 1.4
**Urinalysis**								
Volume (mL)	10.11 ± 19.51	3.26 ± 1.87	3.41 ± 2.72	9.09 ± 20.41	2.68 ± 3.30	1.49 ± 1.13	2.56 ± 2.49	2.53 ± 1.83
Specific gravity	1.0436 ± 0.0247	1.0475 ± 0.0128	1.0625 ± 0.0407	1.0544 ± 0.0242	1.0564 ± 0.0254	1.0781 ± 0.0336	1.0518 ± 0.0263	1.0539 ± 0.0256
pH	6.40 ± 0.39	6.30 ± 0.42	6.35 ± 0.41	6.45 ± 0.37	5.95 ± 0.50	5.90 ± 0.32	5.61 ± 0.33	5.95 ± 0.37

A/G = albumin/globulin; ALP = alkaline phosphatase; ALT = alanine aminotransferase; AST = aspartate aminotransferase; G3P-01 = rhamnogalacturonan-I-enriched pectin extract; GGT = *gamma*-glutamyltransferase; HDL = high-density lipoprotein; LDL = low-density lipoprotein; ppm = parts per million; SD = standard deviation.

^a^Significantly different from control (Kruskal-Wallis & Dunn, *P* ≤ 0.05; ^****^P ≤ 0.01).

^b^Significantly different from control (Anova & Dunnett, *P* ≤ 0.05; ^**^P ≤ 0.01).

#### Hematology

There were no significant test article-induced changes in the hematological parameters analyzed in this study (Table 6). Red blood cells, white blood cells, and differential counts, platelets counts, as well as hemoglobin and related parameters (e.g. corpuscular hemoglobin concentration), were unaffected by exposure to G3P-01. While there was no significant effect in lymphocyte count in any of the male and female dose groups until Day 93 of the study, there was a significant (*P* < 0.05) reduction in lymphocyte count in male rats in the high-dose recovery group on Day 122 relative to study commencement (See supplementary data). In male rats that received 36,000 ppm G3P-01, statistically significant (*P* < 0.01) increases in APTT times were reported on Day 93 in comparison with the start of the study (Table 6). In addition, statistically significantly prolonged mean APTT and higher mean fibrinogen values were noted in the 36,000 ppm G3P-01 group males on Day 121 when compared to the control group (See supplementary data). However, all changes were within the normal limits of the laboratory historical database range.^3^

**Table 6:** Results of hematological analysis in Sprague-Dawley rats administered G3P-01 in the diet for 13 weeks.

Parameter (mean ± SD)	Dose group (ppm)							
	Males				Females			
	0 (control)	9,000	18,000	36,000	0 (control)	9,000	18,000	36,000
White blood cells (10^3^/μL)	8.69 ± 2.33	9.25 ± 1.16	10.08 ± 2.13	8.28 ± 1.51	6.82 ± 1.96	6.10 ± 1.24	6.00 ± 0.80	5.62 ± 1.58
Neutrophils (10^3^/μL)	2.03 ± 0.98	1.73 ± 0.45	1.65 ± 0.40	1.29 ± 0.39	1.17 ± 0.35	1.14 ± 0.48	1.09 ± 0.36	0.93 ± 0.24
Lymphocytes(10^3^/μL)	6.10 ± 1.64	6.94 ± 1.45	7.83 ± 1.90	6.43 ± 1.28	5.21 ± 1.74	4.56 ± 0.84	4.50 ± 0.57	4.35 ± 1.40
Monocytes (10^3^/μL)	0.31 ± 0.11	0.32 ± 0.07	0.31 ± 0.10	0.30 ± 0.11	0.22 ± 0.08	0.21 ± 0.08	0.25 ± 0.06	0.18 ± 0.05
Eosinophils (10^3^/μL)	0.13 ± 0.04	0.16 ± 0.03	0.15 ± 0.07	0.14 ± 0.04	0.13 ± 0.09	0.12 ± 0.10	0.09 ± 0.04	0.11 ± 0.09
Basophils (10^3^/μL)	0.023 ± 0.012	0.028 ± 0.008	0.033 ± 0.015	0.019 ± 0.007	0.015 ± 0.008	0.013 ± 0.007	0.013 ± 0.005	0.010 ± 0.005
Large unstained cells (10^3^/μL)	0.10 ± 0.05	0.08 ± 0.04	0.11 ± 0.05	0.10 ± 0.05	0.070 ± 0.063	0.064 ± 0.027	0.061 ± 0.020	0.050 ± 0.023
Red blood cells (10^6^/μL)	8.50 ± 0.36	8.66 ± 0.24	8.81 ± 0.41	8.64 ± 0.30	8.35 ± 0.59	8.50 ± 0.43	8.29 ± 0.36	8.31 ± 0.31
Hemoglobin (g/dL)	15.57 ± 0.47	15.79 ± 0.61	15.82 ± 0.35	15.79 ± 0.43	15.15 ± 0.69	15.16 ± 0.56	15.00 ± 0.65	14.98 ± 0.48
Hematocrit (%)	47.69 ± 1.57	48.03 ± 1.84	48.62 ± 1.37	48.60 ± 1.23	46.19 ± 2.17	46.45 ± 1.81	46.30 ± 1.86	45.81 ± 1.30
Mean corpuscular volume (fL)	56.18 ± 1.75	55.49 ± 1.38	55.24 ± 2.40	56.29 ± 2.14	55.44 ± 1.77	54.72 ± 1.25	55.83 ± 0.74	55.15 ± 1.39
Mean corpuscular hemoglobin (pg)	18.33 ± 0.61	18.23 ± 0.68	17.97 ± 0.79	18.26 ± 0.63	18.17 ± 0.60	17.84 ± 0.46	18.10 ± 0.36	18.05 ± 0.53
Mean corpuscular hemoglobin concentration (g/dL)	32.65 ± 0.56	32.83 ± 0.66	32.53 ± 0.34	32.46 ± 0.43	32.77 ± 0.38	32.64 ± 0.33	32.38 ± 0.34	32.70 ± 0.40
Red blood cell distribution width (%)	12.23 ± 0.37	12.40 ± 0.33	12.25 ± 0.55	12.55 ± 0.52	11.52 ± 0.53	11.68 ± 0.40	11.64 ± 0.56	11.35 ± 0.39
Platelets (10^3^/μL)	924.2 ± 102.0	979.9 ± 124.0	956.2 ± 118.7	959.3 ± 61.8	916.2 ± 98.0	929.8 ± 164.0	968.6 ± 67.9	986.7 ± 116.4
Reticulocytes (10^9^/L)	192.3 ± 35.2	193.3 ± 29.9	182.6 ± 32.1	189.9 ± 22.8	183.7 ± 36.5	174.7 ± 33.6	188.0 ± 23.9	190.9 ± 43.3
**Blood coagulation analysis**								
PT (seconds)	16.30 ± 0.70	16.39 ± 0.79	16.55 ± 0.53	17.52 ± 1.79	15.28 ± 0.57	15.48 ± 0.72	15.51 ± 0.67	5.10 ± 0.39
APTT (seconds)	14.96 ± 1.29	15.46 ± 1.11	16.11 ± 1.68	17.13 ± 1.72^a^	13.26 ± 1.24	13.02 ± 1.17	14.06 ± 2.21	14.01 ± 1.20
Fibrinogen (mg/dL)	364.9 ± 33.7	362.1 ± 30.5	349.3 ± 28.7	339.1 ± 39.0	235.2 ± 55.9	229.5 ± 58.9	261.3 ± 42.4	227.1 ± 26.8
**Thyroid hormone analysis**								
T3 (pg/mL)	496.3 ± 117.5	452.9 ± 93.4	404.1 ± 64.1	368.6 ± 66.7^a^	633.4 ± 133.4	702.3 ± 90.8	555.7 ± 94.7	553.6 ± 79.0
T4 (ng/mL)	56,310.0 ± 10,755.2	53,855.6 ± 8175.6	58,390.0 ± 5567.1	51,550.0 ± 5553.6	37,550.0 ± 9250.6	44,800.0 ± 10,702.2	37,950.0 ± 11,612.0	34,720.0 ± 7452.6
TSH (pg/mL)	2546.400 ± 1397.890	3301.100 ± 1176.960	2580.400 ± 1736.966	3434.400 ± 1360.981	1121.300 ± 567.530	1001.000 ± 551.463	1144.400 ± 576.944	1554.500 ± 847.438

APTT = activated partial thromboplastin time; G3P-01 = rhamnogalacturonan-I-enriched pectin extract; PT = prothrombin time; SD = standard deviation; T3 = triiodothyronine; T4 = thyroxine; TSH = thyroid stimulating hormone.

^a^Significantly different from control (Anova & Dunnett, *P* ≤ 0.01).

There was a significant (*P* < 0.01) reduction in T3 levels in male rats at the top dose relative to study commencement (Table 6). These changes were mild and without any corresponding changes in total T4 or TSH levels, and there were no corresponding adverse microscopic findings.

#### Pathology

No test substance-related macroscopic findings were reported at the end of the dosing period. However, at the end of the recovery period, significant enlargement of the pituitary gland was observed in 2 females in the high-dose group compared to the control group; however, the mean pituitary gland weights were within historical ranges of the testing laboratory.^4^ No corresponding microscopic changes were observed. No other significant test article-related macroscopic or microscopic changes were observed in the animals. Therefore, any microscopic findings observed in this study were deemed incidental and normal for the tested rat strain and age. Further, the incidence of any observed effects was similar between the control group and the groups that received G3P-01 in the diet.

## Discussion

The present study battery— bacterial mutation test, in vitro micronucleus assay, 13-week repeat-dose toxicity study—was designed to evaluate the safety of G3P-01, a pectin-derived extract from pumpkin (*C. moschata* var. Dickinson). All studies were conducted in accordance with applicable OECD test guidelines. Two representative batches of G3P-01 were used in the studies. G3P-01 remained stable beyond the study period as shown by the concentration of the pectin markers arabinose, galactose, uronic acids, and NSPs. Therefore, results from these studies are indeed from the test material and not any possible impurities or degradation products.

The bacterial mutagenicity study was conducted to evaluate the potential of the test material to induce genetic damage that results in gene mutation.^37^^,^^43^ A positive test result is an indication of the mutagenicity of the test material and its likelihood to be a carcinogen, as >50% of carcinogens have tested positive in Ames mutagenicity test.^44^ The results from the bacterial reverse mutation test demonstrate that G3P-01 is non-mutagenic both in the presence and absence of metabolic bioactivation. This is consistent with the conclusion of the European Food Safety Authority, who noted that no genotoxic activities have been observed for pectins,^45^ as well as the findings associated with other studies of pectins and dietary fibers whereby no mutagenicity was detected in the concentrations of the tested materials.^46^ Similarly, results from the in vitro micronucleus assay indicate that the test article was non-cytotoxic in culture. Further, there was no statistically significant difference in the number of micronucleated cells in G3P-01–incubated cells in comparison with the negative control. Data from the positive and negative controls were in line with historical data,^5^ indicating that the test system was fully functional. The absence of a positive result in the micronucleus assay indicates that G3P-01 is non-clastogenic.^47^ This result is consistent with the findings reported by Borzelleca et al.,^46^ who demonstrated that sodium pectate was non-clastogenic. Jonker et al.^23^ tested carrot pectin in a battery of genetic toxicity studies, including an in vitro micronucleus assay, and reported a negative result. The absence of mutagenicity in the bacterial mutagenicity study and absence of clastogenicity in the in vitro micronucleus study in mammalian cells demonstrated that G3P-01 does not have carcinogenicity potential similar to other pectins and dietary fibers in general. In contrast, it has been suggested that pectins have the potential to prevent cancers.^48^^,^^49^

In the repeat-dose study, G3P-01 was well tolerated, as there were no adverse effects or mortality reported. There was, however, yellow coloration of the feces in the treated group. *Cucurbita* species contain large amounts of fat-soluble carotenoids, primarily *beta*-carotene.^50^ Carotenoids are known to cause discoloration of feces without toxicological effects.^51^ As G3P-01 contains a considerable amount of fat and has a bright orange pigment, the color of the feces was attributed to the orange color of G3P-01 in the diet.

In the repeat-dose study, significantly higher cecum weights were reported in female rats that received 18,000 ppm G3P-01 in the diet and males and female rats in the 36,000 ppm G3P-01 group, with the latter persisting in the recovery phase. This cecal weight increase was not correlated with microscopic findings and would not be considered adverse. Similar findings with relatively higher cecal weights have been reported in other dietary studies in rats fed dietary fiber.^23^^,^^52–54^ Enlargement of the cecum typically occurs following consumption of a large amount of non-digestible carbohydrates. Similar fermentable pectins and dietary fiber in general are known to increase the production of SCFAs, which are rapidly absorbed from the large intestine.^55–57^ Chronic and/or acute exposure to SCFAs has been demonstrated to result in enhancement of epithelial cell proliferation,^58^ thereby possibly resulting in the increase in cecal weight observed in the current study. Other mechanisms, such as increased osmolarity of cecal content or an increase in bulk content, can also play a role in the effect of G3P-01 on cecal weight.^23^^,^^59^ The latter findings indicated that these gastrointestinal effects are a normal response to consumption of dietary fibers. Therefore, the increase in cecal weight is considered non-adverse and of no toxicological implication.^60^

Significant reduction in cholesterol was recorded in male rats that received 18,000 and 36,000 ppm G3P-01 after the 13-week feeding period and upon recovery (Day 122). There was a corresponding reduction in LDL in male rats fed 36,000 ppm G3P-01. Dietary fibers have been shown to have a beneficial effect in reducing plasma cholesterol, with a potential downstream effect on cardiovascular diseases and atherosclerosis.^61^^,^^62^ SCFAs produced by the intestinal microflora upon ingestion of dietary fibers have a regulatory function in the cellular metabolism of fatty acids directly and at a genetic level.^63^ Butyrate, a SCFA, has been described to reduce intestinal absorption of total cholesterol by regulating the expression of transporters such as Niemann-Pick C1-like 1 (Npc1l1; a major intestinal cholesterol transporter) and ATP-binding cassette (ABC).^64^ Also, SCFAs have been shown to significantly reduce plasma total cholesterol by excretion of bile in the feces and up-regulation of the sterol-regulatory element-binding protein 2 (SREBP2), LDL receptor, and cholesterol-specific cytochrome P450 7A1 (CYP1A7) in the liver.^65^ This and other possible mechanism by which SCFAs can reduce total cholesterol have been reviewed by Nogal et al.^66^ Consequently, human clinical trials, such as those reported by Haghikia et al.,^67^ indicate a reduction of LDL and non-HDL. In male rats of the 36,000 ppm G3P-01 group, there was a significantly (*P* < 0.01) lower level of T3 compared with the control group. A reduction of T3 levels is indicative of underactive thyroid or hypothyroidism,^68^ and this is associated with non-specific signs such as fatigue, constipation, and muscle cramps, among others.^69^ These clinical signs were not reported in any of the study animals. Gross pathology observations of the thyroid glands did not indicate any test article-related effects. Hence, the change in T3 is considered non-adverse and of no toxicological significance. Furthermore, there were no statistically-significant changes in other biochemical parameters, such as those relating to hepatic function, renal function, as well as electrolytes. The lack of significant changes in these parameters is supported by the absence of histological changes in the liver, kidney, and other essential organs collected throughout the course of the study.

Although all hematological parameters were unaffected at the end of the 13-week dosing period, there was a significant increase in lymphocyte count in male rats of the high-dose group when compared with the control group (See supplementary data). There was also a significant increase in APTT in male rats of the high-dose group. However, the values obtained—both for the lymphocyte count and the APTT—were within in-house historical control values for the strain and age of rats employed in this study.^6^ Consequently, these effects are deemed incidental, unrelated to exposure to G3P-01, and of no toxicological relevance.

In summary, data from the current study on G3P-01 indicates that G3P-01 is non-genotoxic and not orally toxic to rats at the doses tested. This is in line with the safety of dietary fibers, which have been extensively shown to be safe upon oral ingestion. Pectins, the class of dietary fiber contained in G3P-01, have a substantial history of consumption in humans; they are composed of polysaccharides that are not absorbed in the gastrointestinal tract.^70^ These polysaccharides are broken down by intestinal microbiota and converted to SCFAs, which contribute to a host of physiological benefits as described above.^61^^,^^62^ Due to their innocuous nature, dietary fibers do not have a no-observed-adverse-effect level (NOAEL) or an acceptable daily intake level. G3P-01 is safe, as supported by the history of consumption of pumpkins and dietary fiber. Consequently, the studies performed as part of this evaluation support the safe use of G3P-01 as a food ingredient and dietary supplement.

## Conclusions

This safety evaluation demonstrates that G3P-01 is non-genotoxic, as study results were negative in an Ames study and in vitro micronucleus assay. Further, in a 13-week repeat-dose oral toxicity study, there were no mortalities and no test substance-related effects in body weight, food consumption, neurobehavioral assessments, hematology, blood biochemistry, coagulation, or urinalysis. Further, there were no test substance-related effects on ophthalmic, estrous cycle, macroscopic, or microscopic findings. Overall, these results indicate that G3P-01 does not induce adverse effects at the tested intake levels. As a result, the subchronic NOAEL for G3P-01 is the highest tested concentration of 36,000 ppm, equivalent to 1,899 mg/kg body weight/day in male rats and 2,361 mg/kg body weight/day in female rats.

## Supplementary Material

Supplementary_tables_tfae004

## Data Availability

The data used are confidential.

## Footnotes

1 See footnote “e” of Table 2 for historical data.

2 Weight on day 122 was 325.4 ± 29.2 mg in the 36,000-ppm group versus 389.8 ± 43.9 mg in the control group.

3 Historical control values for APTT and fibrinogen for Crl:CD(SD) rat as reported by the testing laboratory are as follows: APTT (Males: 11.5–18.1 s; Females: 9.8–15.6 s) and fibrinogen (Males: 347–582 mg/dL Females: 190–295 mg/dL).

4 Historical value for absolute organ weight for pituitary Crl:CD(SD) rats is as follows: Male (0.01–0.0217 g) and female (0.0081–0.0397 g)

5 See footnote “e” of Table 2 for historical data.

6 Historical control values for APTT and fibrinogen for Crl:CD(SD) rat as reported by the testing laboratory are as follows: APTT (Males: 11.5–18.1 s; Females: 9.8–15.6 s) and fibrinogen (Males: 347–582 mg/dL Females: 190–295 mg/dL).
