# Supplementary material for: Toxicological evaluation of a pumpkin-derived pectin preparation: in vitro genotoxicity studies and a 13-week oral toxicity study in Sprague-Dawley rats
Source: Toxicol Res (Camb). 2024 Jan 23;13(1):tfae004. doi: 10.1093/toxres/tfae004 (PMC10807847; doi:10.1093/toxres/tfae004)
Supplement: Supplementary_tables_tfae004 [file supplementary_tables_tfae004.docx]

| **Table 1**. Hematological analysis in recovery Sprague-Dawley Rats 28 days after G3P-01 diet administration  for 13 weeks. | | | | | |
| --- | --- | --- | --- | --- | --- |
| **Parameter**  **(mean ± SD)**  **Day 122** | **Dose group (ppm)** | | | | |
|  | **Males** | | | **Females** | |
|  | **0 (control)** | **36,000** | **0 (control)** | | **36,000** |
| White blood cells (10^3^/µL) | 8.86 ± 1.43 | 6.99 ± 1.52 | 4.06 ± 0.83 | | 3.72 ± 1.17 |
| Neutrophils (10^3^/µL) | 1.93 ± 0.57 | 2.03 ± 0.56 | 0.97 ± 0.60 | | 0.59 ± 0.25 |
| Lymphocytes(10^3^/µL) | 6.37 ± 1.10 | 4.30 ± 1.06* | 2.74 ± 0.48 | | 2.81 ± 1.30 |
| Monocytes (10^3^/µL) | 0.34 ± 0.11 | 0.36 ± 0.07 | 0.21 ± 0.08 | | 0.17 ± 0.06 |
| Eosinophils (10^3^/µL) | 0.18 ± 0.03 | 0.18 ± 0.03 | 0.86 ± 0.15 | | 0.78 ± 0.23 |
| Basophils (10^3^/µL) | 0.01 ± 0.00 | 0.008 ± 0.004 | 0.008 ± 0.004 | | 0.010 ± 0.007 |
| Large unstained cells (10^3^/µL) | 0.12 ± 0.05 | 0.011 ± 0.054 | 0.044 ± 0.015 | | 0.06 ± 0.033 |
| Red blood cells (10^6^/µL) | 8.56 ± 0.38 | 8.54 ± 0.65 | 7.89 ± 0.35 | | 7.67 ± 0.44 |
| Hemoglobin (g/dL) | 15.44 ± 0.69 | 15.94 ± 1.17 | 14.52 ± 0.40 | | 13.96 ± 1.06 |
| Hematocrit (%) | 47.58 ± 1.89 | 48.78 ± 3.48 | 43.60 ± 1.46 | | 41.54 ± 2.65 |
| Mean corpuscular volume (fL) | 55.64 ± 1.32 | 57.14 ± 2.66 | 55.26 ± 2.98 | | 54.22 ± 0.70 |
| Mean corpuscular hemoglobin (pg) | 18.06 ± 0.63 | 18.66 ± 0.81 | 18.40 ± 0.80 | | 18.20 ± 0.57 |
| Mean corpuscular hemoglobin concentration (g/dL) | 32.48 ± 0.39 | 32.70 ± 0.50 | 33.34 ± 0.43 | | 33.56 ± 0.65 |
| Red blood cell distribution width (%) | 13.56 ± 0.34 | 12.68 ± 0.59* | 12.52 ± 0.43 | | 12.28 ± 0.56 |
| Platelets (10^3^/µL) | 802.6 ± 108.5 | 552.4 ± 78.5 | 931.2 ± 20.0 | | 215.5 ± 32.23 |
| Reticulocytes (10^9^/L) | 198.44±28.31 | 212.98±42.17 | 1024 ± 64.4* | | 204.82 ± 21.4 |
| **Blood coagulation analysis** | | | | | |
| PT (seconds) | 15.66 ± 0.67 | 15.66 ± 0.65 | 15.38 ± 0.31 | | 15.68 ± 0.68 |
| APTT (seconds) | 14.14 ± 1.69 | 16.40 ± 1.11* | 13.78 ± 1.00 | | 14.60 ± 0.74 |
| Fibrinogen (mg/dL) | 364.0 ± 22.9 | 440.2 ± 62.7* | 242.6 ± 20.8 | | 238.8 ± 23.6 |
| APTT = activated partial thromboplastin time; G3P-01 = rhamnogalacturonan-I–enriched pectin extract; PT = prothrombin time; SD = standard deviation.  * Signiﬁcantly different from control (Anova & Dunnett, p≤0.01). | | | | | |
